# 

**Published:** 1873-07

**Authors:** 


### SOUTHERN DENTAL SOCIETY.

The next annual meeting of the Southern Dental Association will be held in the city of Baltimore, Md., the last Tuesday in July, 1873, commencing at 10 o'clock A. M.

It is sincerely hoped and expected, that the members of the following committees will correspond with each other, so as to act in concert, and furnish a report on each subject, and thereby make our next meeting one of the most interesting and instructive ever held, as it is expected to be the largest

### OFFICERS.

President—Dr. H. M. Grant, Virginia ;

1st Vice President—Dr. F. J. S. Gorgas, Md. ;

2nd Vice President—Dr. T. T. Moore, S. C. ;

3d Vice President—Dr. A. C. Ford, Georgia ;

Corresponding Secretary—Dr. O. J. Bond, S. C. ;

Recording Secretary—Dr. J. F. Thompson, Virginia ;

Treasurer—Dr. H. A. Lawrence, Georgia.

Executive Committee—Drs. W. H. Thackston, John Mahony, Richmond ; F. J. S. Gorgas. A. G. Bouton, Maryland ; A. C. Ford, Ga.

Membership—H. J. Royal, Ga. ; O. J. Bond, South Carolina ; James Johnston, Virginia.

Publications—Drs. F. J. S. Gorgas, Maryland ; James F. Thompson, Virginia ; H. A. Lawrence, Georgia.

Dental Education—Drs. E. Floyd, North Carolina ; Judson B. Wood, Virgina ; James Knapp, Louisiana.

Physiology and Surgery—Drs. S. P. Cutler, J. C. McAuley Alabama ; S. A. White, Georgia.

Histology and Microscopy—Drs. Wm. H. Atkinson, B. F Arrington, North Carolina ; E. M. Allen, Georgia.

Dental Chemistry—Drs. George H. Winkler, Georgia ; John G. Angell, Louisiana ; Samuel Rambo, Alabama.

Dental Therapeutics—Drs. G. T. Baker, Philadelphia, Pa ; W. C. Wardlaw, H. J. Royal, Georgia.

Operative Dentistry—Drs. F. Y. Clark, Georgia ; S. J. Cobb, Tenn. ; T. T. Moore, South Carolina.

Mechanical Dentistry—Drs. S. G. Holland, Georgia ; G. F. Keiser, Virginia ; E. A. Herman, Tennessee.

Dental Literature—Drs. G. F. S. Wright, T. B. Legare, J. B. Patrick, South Carolina.

Voluntary Essays—Drs. W. H. Shadoan, Kentucky; Wm. H. Burr, Georgia; J. W. Scribner, Virginia.

Dental Appliances and Improvements—Drs. F. A. Jeter, Virginia. A. F. Bignon, Ga; G. W. Peasants, Va.; G. W. McIlheny, Ga.

JAMES F. THOMPSON, M. D., D. D. S.
*Recording Secretary*,

### DENTAL CONVENTION.

NEW YORK JUNE, 20th, 1873.

MR. EDITOR.—The Committee of Arrangements would respectfully inform the members of the Profession, that the Nineteenth Annual Meeting of the American Dental Convention, will be held at *Saratoga Springs*, on the 12th day of August, (second Tuesday,) commencing at 10 A. M.

*Messers Bresslin, Gardner & Co.* of the GRAND UNION HOTEL, have generously tendered their *large Hall*, rooms for committees &c, also their *splendid parlors*, *grounds and band of music*, for our social entertainment, etc.

*The town authorities*, have also offered us a very suitable room for our meetings, in the new and magnificent Hall recently erected there.

*The committee* have made arrangements for the display and exhibition of Dental materials, and appliances of all kinds, also, for clinical operations during the entire session.

*Dentists and others* desirous of exhibiting instruments, materials or improvements in any department of Dentistry, are particularly invited to present them as early in the session as possible, and to notify the committee of their intention to do so.

As August is a very busy month with hotel keepers, it will be advisable for those intending to be present, to notify the committee of their wishes, etc.

The committee will see that good accommodations are provided for all who give timely notice, and for others as far as possible, and will endeavor to make this meeting a pleasant and profitable gathering to all.

The profession generally, are cordially invited to be present.

J. G. AMBLER.

Chairman of Committee, 25. W. 23rd. Street, N. Y.

### NEW JERSEY STATE DENTAL SOCIETY.

The third annual meeting of this Society will commence on Tuesday, July 6th, 1873, at 10 o'clock, A. M., at the United States Hotel, Long Branch.

FIRST DAY, TUESDAY.

President's Address, and the transaction of business pertaining to the Society, including the election of officers.

SECOND DAY, WEDNESDAY.

Clinic, by Dr. W. Pinney, of Newark.

Subjects for Discussion.—10½ o'clock A. M., Tooth-ache: its Cause and Cure—Dr. C. S. Stockton, of Newark.

11 o'clock, Peridental Inflammation—Dr. W. H. Dibble, of Elizabeth.

11½ o'clock, Transplanting Teeth—Dr. L. H. De Lange, of Bordentown.

12 o'clock, Capping Nerves—Dr. Fowler, of Newark.

12½ o'clock, Regulating Teeth—Dr. E. F. Hanks, of Jersey City.

AFTERNOON SESSION.

12½ o'clock, Separating Teeth—Dr. Louis Reading, of Trenton.

3 o'clock, Rubber Dam, and appliances—Dr. Cosadi of Jersey City,

3½ o'clock, The Uses of Different Mallets—Dr. Geo. C. Brown, of Mount Holly.

4 o'clock, General Discussion.

THIRD DAY, THURSDAY.

Morning.—Clinics, by Dr. Louis Jack, of Philadelphia; and by Dr. Frank Abbott, of New York City.

AFTERNOON.

2½ o'clock, Mechanical Dentistry.

Arrangements have been made for hotel accommodation. You are particularly requested to attend, and have your name enrolled as a member,

An answer will oblige. Leo. H. De Lange,
*Secretary.*

### THE WISCONSIN STATE DENTAL SOCIETY,

Will be held at the Newhall House, in Milwaukee, on Tuesday and Wednesday, July 8th and 9th, 1873. Arrangements have been made with the propriet ors of the Newhall House, by which the members of the Society will be boarded for $2.00 per day. A large parlor will be furnished for the use of the Society, free of charge.

Members are requested to bring Instruments, and such Specimens as they have, which may be of interest to the profession.

WM. DECKER, S. M. GILMAN, S. E. WADE, Executive Committee.

